# The Function of CBM32 in Alginate Lyase VxAly7B on the Activity on Both Soluble Sodium Alginate and Alginate Gel

**DOI:** 10.3389/fmicb.2021.798819

**Published:** 2022-01-07

**Authors:** Luyao Tang, Enwen Guo, Lan Zhang, Ying Wang, Shan Gao, Mengmeng Bao, Feng Han, Wengong Yu

**Affiliations:** ^1^School of Medicine and Pharmacy, Ocean University of China, Qingdao, China; ^2^Key Laboratory of Marine Drugs, Ministry of Education, Qingdao, China; ^3^Shandong Provincial Key Laboratory of Glycoscience and Glycoengineering, Qingdao, China; ^4^Laboratory for Marine Drugs and Bioproducts of Qingdao National Laboratory for Marine Science and Technology, Qingdao, China

**Keywords:** alginate lyase, alginate gel, insoluble substrate, carbohydrate-binding module (CBM), binding capacity, function

## Abstract

Carbohydrate-binding modules (CBMs), as an important auxiliary module, play a key role in degrading soluble alginate by alginate lyase, but the function on alginate gel has not been elucidated. Recently, we reported alginate lyase VxAly7B containing a CBM32 and a polysaccharide lyase family 7 (PL7). To investigate the specific function of CBM32, we characterized the full-length alginate lyase VxAly7B (VxAly7B-FL) and truncated mutants VxAly7B-CM (PL7) and VxAly7B-CBM (CBM32). Both VxAly7B-FL and native VxAly7B can spontaneously cleavage between CBM32 and PL7. The substrate-binding capacity and activity of VxAly7B-CM to soluble alginate were 0.86- and 1.97-fold those of VxAly7B-FL, respectively. Moreover, CBM32 could accelerate the expansion and cleavage of alginate gel beads, and the degradation rate of VxAly7B-FL to alginate gel beads was threefold that of VxAly7B-CM. Results showed that CBM32 is not conducive to the degradation of soluble alginate by VxAly7B but is helpful for binding and degradation of insoluble alginate gel. This study provides new insights into the function of CBM32 on alginate gel, which may inspire the application strategy of CBMs in insoluble substrates.

## Introduction

Brown algae, important marine primary producers (Enríquez and Borowitzka, 2010), constitute a large amount of biomass in marine ecosystems (Duarte et al., 2004; Roesijadi et al., 2010; Wargacki et al., 2012). Alginate is the most abundant polysaccharide in brown algae, accounting for approximately 40% of its dry weight (Chung et al., 2011; Popper et al., 2011). Alginate is an acidic linear polysaccharide composed of α-L-guluronate (G) and its C5 epimer β-D-mannuronate (M), arranged in three kinds of blocks: homopolymeric M blocks (polyM), homopolymeric G blocks (polyG), and heteropolymeric blocks composed of alternating M and G (polyMG) (Gacesa, 1988). Due to its high safety, unique physical and chemical properties, and biological functions, alginate has a wide range of applications in the fields of food, agriculture, and medicine (Stevens et al., 2004; Aarstad et al., 2019; Liu et al., 2019). Studies have found that alginate can spontaneously form a gel in the presence of divalent cations (Il Kim et al., 2021). Alginate gel has received extensive attention in the field of tissue engineering due to its excellent biocompatibility and controllable release characteristics as a carrier of living cells (Nguyen and Anker, 2014). In addition, as a tough, elastic, non-thermally reversible colloidal material, alginate gel is favored in agriculture and food fields because of its use to encapsulate metabolites such as endosulfan, curcumin, and astaxanthin (Bozanic et al., 2009; Song et al., 2012, 2020). However, the slow and uncontrollable degradation of alginate gel has become the main factor limiting its development (Kunjukunju et al., 2018). Compared with non-enzymatic degradation, enzymatic degradation is less stimulating to cells and can control the release rates of small molecular compounds, which has become a hot spot in current research.

Among the thousands of alginate lyases that have been discovered, only a few can degrade alginate gel, such as alginate lyase (AL) from *Sphingobacterium multivorum* (Nguyen and Anker, 2014) and alginate lyase from *Flavobactierium* (Kunjukunju et al., 2018). Alginate lyases are polysaccharide lyases that specifically degrade alginate and mainly come from marine microorganisms, marine mollusks, and brown algae (Wong et al., 2000; Ertesvag, 2015). They degrade the 1,4-glycosidic bond through a β-elimination reaction to form an unsaturated double bond between C4 and C5, generating a 4-deoxy-L-*erythro*-hex-4-enopyranosyluronic acid at the non-reducing end (Preiss and Ashwell, 1962a). Alginate lyases are important in the marine carbon cycle (Matos et al., 2016) and have been widely applied in biochemistry, medicine, and energy (Chen et al., 2017; Tang et al., 2020; Zhao et al., 2020). Based on substrate specificity, alginate lyases are classified as polyM-specific (EC 4.2.2.3), polyG-specific (EC 4.2.2.11), and bifunctional, which can degrade both polyM and polyG (EC 4.2.2.-). According to sequence similarities, alginate lyases are classified into 13 polysaccharide lyase (PL) families (PL5, 6, 7, 14, 15, 17, 18, 31, 32, 34, 36, 39, and 41) in the Carbohydrate-Active enZYmes (CAZy) database^1^ (Lombard et al., 2014).

With the deepening of research, it has been discovered that in addition to catalytic domains (CD), some multimodule alginate lyases have non-catalytic regions (NCRs). The carbohydrate-binding module (CBM), as an important type of NCR, is currently further subdivided into 88 different CBM families in the CAZy database. CBMs do not have the catalytic ability but may have various effects on enzyme characteristics, such as targeting substrates (Lyu et al., 2018), disrupting insoluble substrate structures (Bernardes et al., 2019), or regulating activity (Zhang et al., 2019). Among them, CBM_4_9, CBM13, CBM16, and CBM32 are the most common alginate lyases. Although studies have shown that CBMs in alginate lyases influence enzyme activity, substrate-specific recognition and product distribution of soluble alginate, such as CBM_4_9 from Aly1 (Cheng et al., 2017), CBM13 from AlyL2 (Li et al., 2015), and CBM32 from TsAly7B (Zhang et al., 2019), the effect of CBMs on alginate gel is still unclear.

In this study, we screened the high-producing alginate lyase *Vibrio xiamenensis* QY104. The fermentation broth was purified to obtain the native enzyme VxAly7B. Temperature and time were the main factors that affected the spontaneous cleavage of VxAly7B. Through genome sequencing and analysis, VxAly7B was found to include CBM32 and PL7 catalytic domains. To explore the function of CBM32 in depth, we cloned and purified the full-length sequence of VxAly7B (VxAly7B-FL) and two truncated mutants: VxAly7B-CM (PL7) and VxAly7B-CBM (CBM32). The results show that CBM32 plays an important role in the characteristics of VxAly7B, especially in the degradation of alginate gel. This study is an important exploration of the effect of CBM32 on alginate gel, provides new ideas and possibilities for the further application of alginate gel and expands the application of insoluble substrates in production.

## Materials and Methods

### Bacterial Strains, Plasmids, and Chemicals

The bacterium *V. xiamenensis* QY104, isolated from Qingdao coastal seawater, was cultured at 25°C in medium (pH 7.0) containing (w/v) 2.5% NaCl, 0.3% sodium alginate, 0.25% casamino acids, 0.5% MgSO_4_⋅7H_2_O, 0.1% KCl, 0.02% CaCl_2_, 0.15% NaH_2_PO_4_, 0.2% NaNO_3_, and 0.002% FeSO_4_⋅7H_2_O (1.5% agar in solid medium). The bacterial genome was sequenced and assembled by Novogene Bioinformatics Technology Co., Ltd. (Tianjin, China).

*Escherichia coli* DH5α was used for DNA cloning. *E. coli* BL21 (DE3) and pET-24a (+) plasmids (Takara Co., Ltd., Dalian, China) were used for recombinant protein expression. Chemical reagents were all analytical grade. Molecular cloning was performed using standard procedures (Green and Sambrook, 2012). Sodium alginates were purchased from Sigma-Aldrich (St. Louis, MO, United States). PolyM and polyG were obtained from Qingdao BZ Oligo Biotech Co., Ltd. (Qingdao, China).

### Screening of Alginate-Degrading Bacterial Strains

To obtain bacterial strains with alginate degradation activity, we used agar solid mediums with sodium alginate as the sole carbon source to isolate the bacteria from four seawater samples of Qingdao coastal seawater at 25°C for 48–72 h. Single colonies were picked up from the plates and further cultivated at 25°C for 48 h in a liquid medium with sodium alginate as the sole carbon source. The culture supernatant was collected after centrifugation at 12,000 r/min for 10 min and used as the crude enzyme solution.

### Enzyme Activity Assay

The β-elimination mechanism of alginate lyases results in double bonds whose accumulation could be monitored by measuring absorbance changes at 235 nm (Wong et al., 2000). Aliquots (0.1 mL) of enzyme solution were added to 0.9 mL of substrate solution containing soluble sodium alginate (0.3% w/v) in 20 mM PB buffer plus 500 mM NaCl at optimum pH and incubated at optimum temperature for 10 min. One unit of enzyme activity was defined as an absorbance increase at 235 nm of 0.1/min.

### Purification of Native VxAly7B

For expression, *V. xiamenensis* QY104 was plated on the abovementioned specific solid medium. Individual colonies were cultured overnight in 5 mL of the corresponding liquid medium at 25°C and 160 r/min. The culture solution was inoculated in the same liquid medium at a ratio of 1% and cultured at 25°C for 12 h. The supernatant after centrifugation at 12,000 r/min was the crude enzyme solution.

In an ice-cold water bath environment, ammonium sulfate powder was slowly added to the crude enzyme solution until the saturation reached 40%. The solution was stirred for 2 h, stranded, and then centrifuged at 12,000 r/min for 30 min. The supernatant after precipitation with ammonium sulfate was purified by fast protein liquid chromatography (FPLC) using a phenyl sepharose fast flow column (GE Healthcare, Stamford, CT, United States) with a flow rate of 2 mL/min. The column was washed with equilibration buffer (2.5 M ammonium sulfate, 50 mM PB, pH 7.0) to remove unbound protein, and a gradient of 20, 40, 60, 80, and 100% elution buffer (50 mM PB, pH 7.0) was applied. The active components were dialyzed into 20 mM PB (pH 7.0). Then, the component was purified using a HiTrap DEAE FF column (GE Healthcare, Stamford, CT, United States) with a flow rate of 1 mL/min. The column was washed with equilibration buffer (20 mM PB, pH 7.0) to remove unbound protein, and a gradient of 20, 40, 60, 80, and 100% elution buffer (1 M NaCl, 20 mM PB, pH 7.0) was applied to purify the native enzyme. The purity and molecular mass of VxAly7B were determined by SDS-PAGE on 12.5% (w/v) resolving gels. Protein concentrations were determined with BCA Protein Quantification Kits (Vazyme Biotech Co., Ltd., Nanjing, China).

### Gel Renaturation and Western Blot of Native VxAly7B

Fermentation broth supernatants at different culture times were collected and subjected to SDS-PAGE. The SDS-PAGE gel was soaked in refolding solution (50 mM Tris-HCl buffer, pH 8.0, 10 mg/mL casein, 2 mM EDTA, 0.01% NaN_3_, 25% methanol) 3 times, each for 30 min. Then, the gel was soaked in rinsing solution (100 mM Tris-HCl buffer, 100 mM NaCl, pH 7.2) 3 times, each for 5 min. Then, the gel was slowly spread on a solidified agarose gel (2% agarose, 0.1% alginate, 1% NaCl, 100 mM Tris-HCl buffer, pH 7.2) and incubated at 30°C for 6 h. After removing the SDS-PAGE gel, 10% cetylpyridinium chloride (CPC) solution was poured on the surface of the solidified agarose gel until a clear transparent circle appeared. Antiserum was prepared by VxAly7B-CM, and Western blot was performed on the above samples.

### Sequence Analysis

Phylogenetic trees were constructed using MEGA 7 via the neighbor-joining method. The sequence of VxAly7B has been submitted to GenBank (accession nos. KC773875 and AGL78596, previously named AlyV4). N-terminal sequencing of the protein was carried out by SCI-LONGS BIOTECH INC. Mass spectrometry-based *de novo* sequencing of intermediate peptides was completed by the National Center for Biomedical Analysis. The amino acid sequence alignment between VxAly7B and crystallized PL7 alginate lyases were obtained using ClustalW and further aligned with ESPript 3.0.^2^

### Cloning, Expression, and Purification of Recombinant VxAly7B and Its Truncated Mutants

Genomic DNA was isolated from *V. xiamenensis* QY104 using a bacterial DNA extraction kit (TIANGEN, Beijing, China). Genome sequencing was performed at Beijing Genomics Institute (BGI, China). Polymerase chain reaction (PCR) amplifications were used to obtain the *vxAly7b* gene without sequence-coding signal peptide. Its truncated mutants were generated, harboring the VxAly7B sequence as the template. The primers are shown in Supplementary Table 1. PCR products were purified using a gel-extraction kit (Vazyme Biotech Co. Ltd., China), digested with *Nde* I and *Xho* I, and ligated to similarly digested pET-24a (+). Primer synthesis and DNA sequencing were performed at Beijing Genomics Institute (BGI, China).

For expression, constructs were transformed into *E. coli* BL21 (DE3) and plated on LB agar containing 30 μg/mL kanamycin monosulfate. Individual colonies were cultured in 5 mL of LB medium with 30 μg/mL kanamycin monosulfate for 16 h at 37°C and 160 r/min. One milliliter of culture was added to 100 mL of LB with 30 μg/mL kanamycin monosulfate and incubated at 37°C and 160 r/min until the OD_600_ reached 0.4–0.6. Expression was induced with 0.1 mM isopropyl 1-thio-β-D-galactopyranoside (IPTG), and incubation then continued at 20°C and 160 r/min for an additional 20 h.

For purification, cells were harvested at 4°C and 12,000 r/min for 20 min. Supernatants were discarded, and pellets were resuspended in 20 mM PB (plus 500 mM NaCl) at pH 7.3 and crushed with a high-pressure crusher (JNBIO, China). Soluble cell lysates were clarified by centrifugation at 12,000 r/min for 20 min at 4°C, and (His)_6_-tagged VxAly7B and its truncated mutants were purified using a HisTrap HP column (GE Healthcare, Stamford, CT, United States). The column was washed with buffer A (20 mM PB, 500 mM NaCl) to remove unbound protein, and a gradient of 5, 20, and 100% buffer B (20 mM PB, 500 mM NaCl, 500 mM imidazole) was applied to purify the recombinant enzymes.

### Enzymatic Kinetic Parameter Assays

To measure the enzyme kinetic parameters of recombinant VxAly7B-FL and recombinant VxAly7B-CM, 0.9 mL of solutions (0.1–10 mg/mL) of alginate in 20 mM PB or Tris-HCl buffer plus 500 mM NaCl was incubated at 40 or 45°C for 10 min. Recombinant VxAly7B-FL and recombinant VxAly7B-CM were added and incubated at optimum temperature for 3 min. *K*_*m*_ and *V*_*max*_ values were determined using the Michaelis-Menten equation, and the curve fitting program was performed by non-linear regression analysis using GraphPad Prism. The turnover numbers (*k*_*cat*_) of the recombinant VxAly7B-FL and recombinant VxAly7B-CM were calculated by the ratio of *V*_*max*_ vs. enzyme concentration.

### Biochemical Characteristics and Spontaneous Cleavage of VxAly7B

Buffers with different pH values, including 50 mM Na_2_HPO_4_-citric acid buffer (pH 3.0–7.0), 50 mM Na_2_HPO_4_-NaH_2_PO_4_ buffer (pH 6.6–7.6), 50 mM Tris-HCl buffer (pH 7.6–8.6) and 50 mM Gly−NaOH buffer (pH 8.6–10.6), were used to measure the optimal pH and the pH stability. The optimal pH was determined with the above buffers. To evaluate pH stability, residual activity was detected after incubating recombinant VxAly7B-FL and recombinant VxAly7B-CM in buffers for 12 h at 4°C. The optimal temperature for enzymatic activity was determined from 0 to 60°C. Thermostability was studied by measuring residual activity after incubation at 0–70°C for 1 h. The effects of metal ions and chelators were examined by monitoring activity in the presence of cations or chelators. NaCl dependence was determined by measuring the activity of recombinant VxAly7B-FL and recombinant VxAly7B-CM with 0.3% (w/v) soluble sodium alginate in 20 mM buffers plus NaCl at different concentrations (0–1.5 mM).

To study substrate specificity, 0.3% (w/v) soluble sodium alginate, polyM, and polyG were used as substrates. To determine the mode of action, 10 mL reaction systems containing 27 mg of sodium alginate and 30 U of recombinant VxAly7B-FL and VxAly7B-CM were allowed to react at 30°C for 0, 1, 5, 10, 30, and 60 min. Changes in the viscosity of degradation products were analyzed by a viscometer. To study the cause of spontaneous cleavage, native VxAly7B and recombinant VxAly7B-FL were incubated at 4, 16, and 25°C for 12, 24, 48, and 60 h and then identified by SDS-PAGE.

### Analysis of End Products by Gel Filtration Chromatography and Negative Ion Electrospray Ionization Mass Spectrometry

The end products of degradation by recombinant VxAly7B-FL and recombinant VxAly7B-CM were prepared by exhaustive degradation with excess activity (10 U of enzyme with 3 mg of alginate). Mixtures were boiled for 10 min to terminate the reactions, filtered through 0.22-μm filters, and centrifuged at 12,000 r/min for 10 min. Supernatants were analyzed by a Superdex peptide 10/300 GL gel filtration column (GE Healthcare, Madison, WI, United States) equilibrated with 0.2 M NH_4_HCO_3_ and detected at 235 nm by FPLC. The flow rate was 0.2 mL/min, and fractions containing uronic acids were pooled, lyophilized, and resuspended in 1 mL of acetonitrile-water (1:1, v/v) mixture. Oligosaccharide samples were analyzed by negative ion ESI-MS with a range from 0 to 1,500 m/z. The scope with no significant product peaks is not shown.

### Native Affinity PAGE

Native affinity PAGE of recombinant VxAly7B-FL and VxAly7B-CM for sodium alginate was qualitatively tested on 10% (w/v) resolving gels as previously described (Arai et al., 2003). Sodium alginate concentrations in running gels were 0.15%. Native affinity polyacrylamide gel was prepared by a native PAGE preparation kit obtained from Sangon Biotech (Shanghai) Co., Ltd., China. Bovine albumin (BSA) was used as the control protein. High molecular weight native electrophoresis protein marker II (45–669 kDa) was obtained from Real-Times (Beijing) Biotechnology Co., Ltd., China.

### Preparation of Alginate Gel Beads

High-viscosity sodium alginate (1%) was dissolved in 20 mM PB (pH 7.0). A total of 200 mL of 0.2 M anhydrous CaCl_2_ solution was prepared and placed in a 500 mL beaker. The beaker was placed on a magnetic stirrer and stirred slowly. A 5 mL syringe (needle 0.7 × 32 mm) was used to suck 3 mL of high-viscosity alginate liquid and drop vertically and slowly at a distance of 10 cm from the liquid surface to make the alginate form a gel bead in the CaCl_2_ solution. Then, the gel beads were slowly stirred for 2 h and washed five times with ddH_2_O. Uniformly sized and round alginate gel beads were immersed in 20 mM PB (pH 7.3 and 500 mM NaCl) for later use.

### Binding of Alginate Gel Beads by Recombinant Enzymes

Since the properties of alginate gel are different from those of soluble substrates, the enzymatic kinetic parameters cannot be determined. Therefore, SDS-PAGE and Langmuir-type adsorption isotherms were selected to determine the binding abilities of the recombinant VxAly7B-FL, VxAly7B-CM, and VxAly7B-CBM to alginate gel.

The same amount of recombinant protein (0.2 nmol) and 10 alginate gel beads were added to EP tubes, and the same molar concentration of BSA was used as a control. The above system was placed at 4°C for 3 h, and the supernatant was collected after centrifugation and identified by SDS-PAGE. At the same time, to quantitatively describe the binding ability of recombinant proteins to insoluble substrates, a Langmuir-type adsorption isotherm binding model was introduced based on Eq. (1). The difference between the protein content in the supernatant before and after incubation was the bound protein content. The different concentrations of recombinant protein and 10 alginate gel beads were added to EP tubes and were placed at 4°C for 3 h. After centrifugation, the protein content in the supernatant was measured by the Bradford method and plotted according to the Langmuir-type adsorption isotherm.


(1) p=adsKpPads,m1+KpP×P(Preiss and Ashwell, 1962b)

Among them, *p*_*ads*_ represents the amount of enzyme adsorbed (mg protein/g substrate), *P* represents the amount of protein added (mg protein/mL), *p_*ads*,m_* represents the maximum adsorption capacity (mg protein/g substrate), and *K*_*p*_ represents the adsorption equilibrium constant (mL/mg protein).

### Degradation of Alginate Gel Beads by Recombinant Enzymes

Alginate gel beads were placed in groups of 10 in a 48-well plate. The same amount of pure enzyme (0.2 nmol) was added to each experimental well. Both control and experimental wells were adjusted with PB to a final volume of 200 μL. The 48-well plate was allowed to stand at 25°C. The morphological changes of the gel beads in each well were observed and recorded every 30 min. At the same time, the supernatant was boiled and centrifuged. The absorbance at 235 nm was measured to characterize the change in reducing sugar content.

## Results

### Isolation and Identification of Bacterial Strain QY104

More than 3,000 bacterial strains were isolated from four seawater samples, of which 12 strains with high enzyme activity reached more than 10 U/mL. Among these strains, strain QY104 had the fastest enzyme production rate and showed high alginate lyase activity, which reached 23 U/mL at 12 h, suggesting that this strain can quickly utilize alginate (Supplementary Figure 1). 16S rRNA gene sequence analysis showed that strain QY104 belongs to *V. xiamenensis*, and the sequence similarity with the closest *V. xiamenensis* G21*^T^* was 98.64% (Supplementary Figure 2).

### Purification and Spontaneous Cleavage of Native Alginate Lyase VxAly7B

**FIGURE 1:**
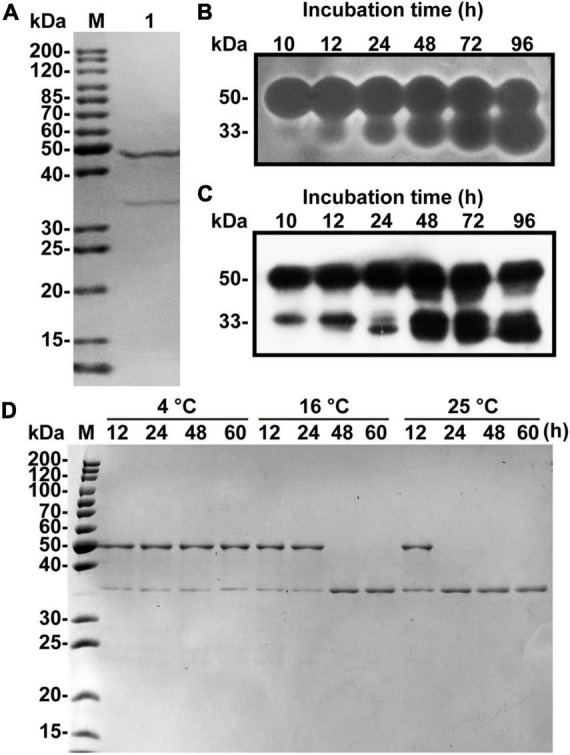
Fermentation, purification and spontaneous cleavage of native VxAly7B. **(A)** Purified native VxAly7B was resolved by 12.5% acrylamide (w/v) SDS-PAGE and stained with Coomassie Blue R-250. Lane M, molecular weight markers; Lane 1, purified native VxAly7B. **(B)** Native VxAly7B production is shown by in-gel renaturation. **(C)** Native VxAly7B production is shown by Western blot. **(D)** Spontaneous cleavage of native VxAly7B was resolved by SDS-PAGE.

A one L culture of *V. xiamenensis* QY104 grown for 12 h was collected for crude enzyme preparation. The three-step purification of the crude enzyme increased the specific activity by 27 times, and the yield was 47.47% (Table 1). The characteristics of native alginate lyase from 12.5% SDS-PAGE showed that it is composed of two peptides with molecular weights of approximately 48 and 34 kDa (Figure 1A). To determine the properties of the two peptides, the production of native enzyme was analyzed by in-gel renaturation during the culture of *V. xiamenensis* QY104. The results showed that with the extension of culture time, the ∼48 kDa band gradually transformed into two bands with alginate lyase activity, which were ∼48 and 34 kDa, respectively. In addition, the ∼34 kDa band gradually increased (Figure 1B). The antiserum was further prepared with native enzyme, and the Western blot results showed that both bands were positive (Figure 1C). Therefore, we speculated that the ∼34 kDa band might come from the ∼48 kDa band.

**TABLE 1:** Purification of native alginate lyase VxAly7B from *V. xiamenensis* QY104.

Step	Total activity (U)	Total protein(mg)	Specific activity (U/mg)	Folds	Recovery (%)
Crude extract	4907.15	101.33	48.43	1.00	100.00
(NH4)_2_SO_4_ Precipitation	4255.50	67.29	63.24	1.31	86.72
Phenyl Sepharose FF	3471.29	7.85	442.20	9.13	70.74
DEAE Sepharose HP	2329.60	1.79	1301.45	26.87	47.47

*1 L of fermentation culture.*

By analyzing the genome sequence of *V. xiamenensis* QY104, we found an open reading frame (ORF) with a length of 1,359 bp, encoding 452 amino acid residues, which was named *vxaly7b*. VxAly7B has a theoretical molecular mass of 47.84 kDa, which is consistent with the size of the above band (∼48 kDa). To verify whether the 34 kDa band comes from VxAly7B, we performed N-terminal sequencing on the 34 kDa band and mass spectrometry-based *de novo* sequencing of the intermediate peptide (Supplementary Table 2). The results showed that the amino acid sequence completely corresponds to the amino acid sequence I155-Y452 of VxAly7B. Based on the above results, it is suggested that the 34 kDa band comes from the spontaneous cleavage of VxAly7B. In addition, we explored the factors of VxAly7B spontaneous cleavage. The results showed that VxAly7B completely cleaves when incubated at 16°C for 48 h or 25°C for 24 h (Figure 1D), suggesting that the extension of time and the rise of temperature would accelerate the cleavage of VxAly7B.

### Sequence Analysis and Heterologous Expression of VxAly7B and Its Truncated Mutants

The isoelectric point (*pI*) of VxAly7B was 4.62. SignalP 5.1 analyses indicated that the signal peptide of VxAly7B contained 21 amino acid residues (Met1 to Ala21). Analyses using the NCBI conserved domain (CD) database indicated that the VxAly7B protein contained an F5_F8_type_C module (Glu28 to Val153) and a putative catalytic module, alginate_lyase2 (Phe175 to His447). F5_F8_type_C was assigned to the CBM32 family according to the CAZy database. Protein sequence alignment showed that the alginate_lyase2 module contained three highly conserved regions of the PL7 family, R(T/S/C/V)EL(G/R)(E/Q), YFKAGXYXQ, and Q(I/V)H (Supplementary Figure 3), and showed the highest sequence identity (51.58%) to alginate lyase AlyA from *Klebsiella pneumoniae* subsp. *aerogenes* among the characterized PL7 alginate lyases.

**FIGURE 2:**
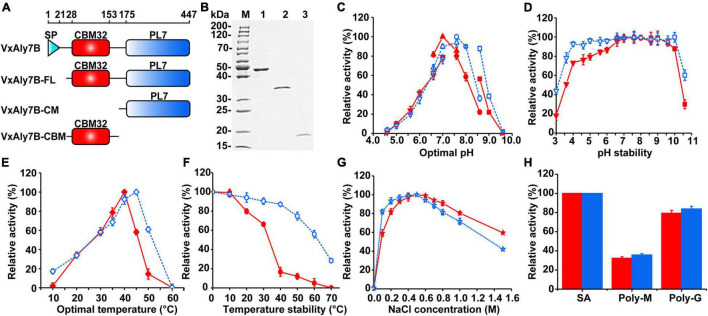
Purification and enzymatic characteristics of recombinant VxAly7B and its truncated mutants. **(A)** Domain structure of full long VxAly7B and its two truncated mutants. **(B)** Purified recombinant VxAly7B-FL, VxAly7B-CM, and VxAly7B-CBM were resolved by SDS-PAGE. Lane M, molecular weight markers; Lane 1, purified recombinant VxAly7B-FL; Lane 2, recombinant VxAly7B-CM; Lane 3, recombinant VxAly7B-CBM. **(C)** Optimum pH for recombinant VxAly7B-FL and VxAly7B-CM were determined by measuring the activity at 40 or 45°C in 50 mM Na_2_HPO_4_-citric acid buffer (pH 4.6–7.0, inverted triangles), 50 mM Na_2_HPO_4_-NaH_2_PO_4_ buffer (pH 6.6–7.6, regular triangles), 50 mM Tris-HCl buffer (pH 7.6–8.6, circles) and 50 mM Gly-NaOH buffer (pH 8.6–9.6, squares). The red line represented the recombinant VxAly7B-FL, and the blue line represented the recombinant VxAly7B-CM. **(D)** To determine pH stability, the residual activity of recombinant VxAly7B-FL and VxAly7B-CM were measured at 40 or 45°C in 20 mM Na_2_HPO_4_-NaH_2_PO_4_ buffer (pH 7.0) and Tris-HCl buffer (pH 7.6) after incubation in 50 mM Na_2_HPO_4_-citric acid buffer (pH 3.0–7.0, inverted triangles), 50 mM Na_2_HPO_4_-NaH_2_PO_4_ buffer (pH 6.6–7.3, regular triangles), 50 mM Tris-HCl buffer (pH 7.6–8.6, circles) and 50 mM Gly-NaOH buffer (pH 8.6–10.6, squares) at 4°C for 12 h, respectively. **(E)** Optimal temperature for recombinant VxAly7B-FL and VxAly7B-CM were determined by measuring the activity at 0–60°C. **(F)** To determine thermostability, the enzyme was incubated at 0–70°C for 1 h. Residual activity was determined at 40 and 45°C, respectively. **(G)** Effect of the NaCl concentration on recombinant VxAly7B-FL and VxAly7B-CM activity. **(H)** Substrate specificity of recombinant VxAly7B-FL and VxAly7B-CM toward alginate, polyM, and polyG.

To investigate the function of CBM32 in VxAly7B, we constructed recombinant plasmids encoding the full-length protein without a signal peptide and two truncated mutants (Figure 2A). Then, the cells were overexpressed in *E. coli* and purified using Ni-Sepharose column chromatography. SDS-PAGE showed apparent molecular masses of 48, 34, and 17 kDa for recombinant VxAly7B-FL, VxAly7B-CM, and VxAly7B-CBM, respectively (Figure 2B), in agreement with the theoretical molecular mass of the recombinant protein fused with the (His)_6_ tag (48.86, 34.12, and 16.61 kDa, respectively). Compared with native VxAly7B, due to the advantages of simple purification steps and short time, we obtained a single band of recombinant VxAly7B-FL. To explore the spontaneous cleavage of recombinant VxAly7B-FL, we incubated recombinant VxAly7B-FL at different temperatures and times. The results showed that recombinant VxAly7B-FL would also spontaneously cleavage between CBM32 and the catalytic domain, similar to the native enzyme (Supplementary Figure 4).

### Enzymatic Characteristics of Recombinant VxAly7B and Its Truncated Mutants

Recombinant enzymes were biochemically characterized using soluble sodium alginate as the substrate. Among them, no activity was detected in recombinant VxAly7B-CBM. Compared to that of recombinant VxAly7B-CM, the *K*_*m*_ value of recombinant VxAly7B-FL was increased 1.15-fold, and the *k*_*cat*_/*K*_*m*_ value was decreased 1.97-fold (Table 2), indicating that CBM32 of VxAly7B is not conducive to the degradation of soluble sodium alginate. The optimum pH of recombinant VxAly7B-FL and VxAly7B-CM were 7.0 and 7.6, respectively (Figure 2C). Recombinant VxAly7B-FL and VxAly7B-CM had broad pH stability range 4.0–10.0 (Figure 2D). In addition, the absence of CBM32 had no obvious effect on the optimum temperatures of recombinant VxAly7B-FL and VxAly7B-CM, which were 40 and 45°C, respectively (Figure 2E). But CBM32 significantly reduced the temperature stability of recombinant VxAly7B-FL. Recombinant VxAly7B-FL retained only approximately 16% of its activity after incubation at 40°C for 1 h, while recombinant VxAly7B-CM retained approximately 87% of its activity. Even after incubation at 60°C for 1 h, recombinant VxAly7B-CM retained 55% of its activity (Figure 2F).

**TABLE 2:** Enzyme kinetic parameters of recombinant VxAly7B-FL and VxAly7B-CM.

	VxAly7B-FL	VxAly7B-CM
*K*_*m*_ (mM)	6.34 ± 0.29	5.49 ± 0.12
*k*_*cat*_ (s^–1^)	1482.71 ± 6.57	2534.89 ± 5.14
*k*_*cat*_*/K*_*m*_ (s^–1^⋅mM^–1^)	234.13 ± 9.93	462.23 ± 3.53

*The K_m_, k_cat_ and k_cat_/K_m_ values presented are means ± standard deviations.*

Considering the influence of NaCl on marine enzyme activity, the enzyme activity in the presence of different NaCl concentrations was measured. The results showed that adding different concentrations of NaCl (0–1.5 M) to the reaction system enhanced the activity of the recombinant VxAly7B-FL and VxAly7B-CM, and the optimal NaCl concentration was 500 mM (Figure 2G), which indicated that the NaCl concentration was relevant only to the catalytic domain. In addition, the results of substrate preference indicated that both recombinant VxAly7B-FL and VxAly7B-CM preferred polyG, whose activities were close to that toward soluble sodium alginate and twice that toward polyM (Figure 2H). The effects of other metal ions, ethylenediaminetetraacetic acid (EDTA) and sodium dodecyl sulfate (SDS), on the activity of recombinant VxAly7B-FL and VxAly7B-CM were also tested, and the results were essentially the same. Ca^2+^ could promote their activities, while other metal ions (Zn^2+^, N^*i2*+^, Cu^2+^, Fe^3+^, etc.), and EDTA and SDS inhibited their activities (Table 3).

**TABLE 3:** Effect of metal ions, chelators, and detergents on recombinant VxAly7B-FL and VxAly7B-CM.

	Concentration (mM)	Relative activity (%) of VxAly7B-FL	Relative activity (%) of VxAly7B-CM
None	–	100	100
LiCl	1	83.64 ± 4.38	95.50 ± 0.48
NH_4_Cl	1	98.44 ± 7.65	93.05 ± 0.24
CuCl_2_	1	11.66 ± 0.81	7.37 ± 0.91
BaCl_2_	1	53.68 ± 3.60	74.75 ± 0.86
ZnCl_2_	1	64.97 ± 1.86	63.30 ± 1.23
MgCl_2_	1	96.71 ± 2.07	94.18 ± 1.60
CaCl_2_	1	111.19 ± 1.93	115.80 ± 2.03
MnCl_2_	1	91.02 ± 0.71	91.46 ± 0.21
NiCl_2_	1	46.96 ± 1.30	74.23 ± 0.96
FeSO_4_	1	75.96 ± 1.68	88.32 ± 4.33
AlCl_3_	1	75.02 ± 6.69	70.26 ± 0.59
SDS	1	38.54 ± 0.04	27.36 ± 2.67
EDTA	1	1.48 ± 0.15	2.27 ± 0.32

**FIGURE 3:**
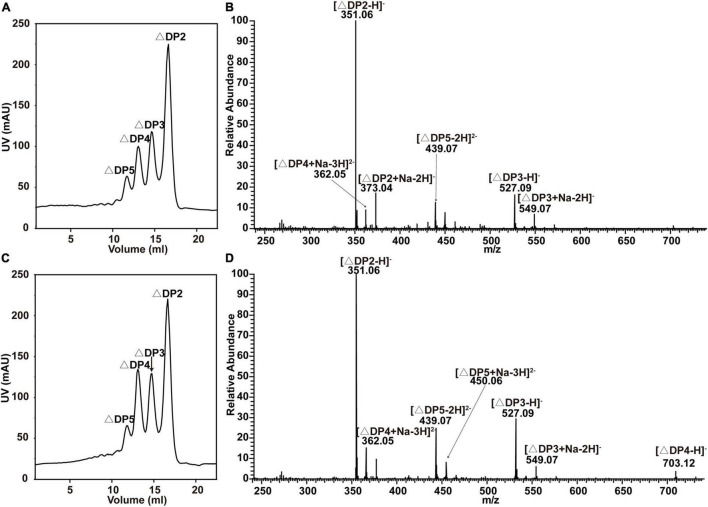
End products of alginate degradation by recombinant VxAly7B-FL and VxAly7B-CM. **(A,B)** End products of recombinant VxAly7B-FL analyzed by gel filtration chromatography and negative ion ESI-MS. **(C,D)** End products of recombinant VxAly7B-CM. Elution volumes were 16.10 mL for unsaturated disaccharides (ΔDP2), 14.90 mL for unsaturated trisaccharides (ΔDP3), 14.10 mL for unsaturated tetrasaccharides (ΔDP4), and 13.44 mL for unsaturated pentasaccharides (ΔDP5).

The modes of action of recombinant VxAly7B-FL and VxAly7B-CM were determined by measuring the viscosity and absorbance at 235 nm of reaction mixtures during degradation. Absorbance at 235 nm increased steadily throughout the entire degradation process; however, viscosity decreased rapidly within the first 10 min and slowly in the latter 50 min (Supplementary Figure 5), suggesting that they act as endo-type alginate lyases. The end products of recombinant VxAly7B-FL and VxAly7B-CM were analyzed by gel filtration chromatography and negative ion ESI-MS. Peak area analysis demonstrated that the molar ratios of the end products (di-, tri-, tetra- and pentasaccharides) by recombinant VxAly7B-FL and VxAly7B-CM were 2.59:1.48:1.27:1 and 2.73:1.87:1.52:1, respectively (Figure 3). The above results indicated that CBM32 does not affect the mode of action and degradation product distribution of VxAly7B.

### Effect of CBM32 of VxAly7B on Insoluble Alginate Gel

**FIGURE 4:**
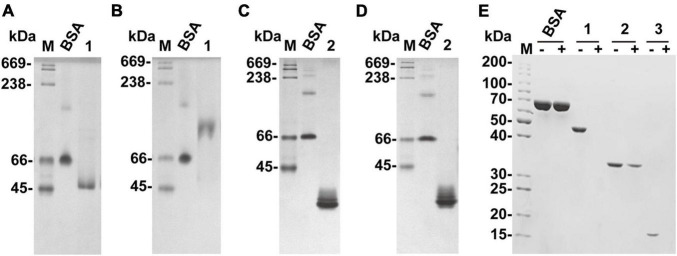
Native affinity PAGE of recombinant VxAly7B-FL and VxAly7B-CM and binding effect of CBM32 on alginate gel beads. **(A,C)** The control gel was a gel without substrate. **(B,D)** Native affinity PAGE was performed using polyacrylamide gels containing sodium alginate. BSA was used as a control protein. Lane M, high molecular weight native electrophoresis protein marker II (45–669 kDa); Lane 1, recombinant VxAly7B-FL; Lane 2, recombinant VxAly7B-CM. **(E)** Binding abilities of recombinant VxAly7B-FL, VxAly7B-CM, and VxAly7B-CBM to alginate gel beads resolved by SDS-PAGE. Lane M, molecular weight markers; Lane 1, recombinant VxAly7B-FL; Lane 2, recombinant VxAly7B-CM; Lane 3, recombinant VxAly7B-CBM.

The affinity of recombinant VxAly7B-FL and VxAly7B-CM with insoluble alginate was qualitatively estimated by native affinity PAGE. The migration of recombinant VxAly7B-FL was significantly slowed in the native affinity PAGE gel containing sodium alginate (Figures 4A,B). The migration of recombinant VxAly7B-CM was also slowed, but to a lesser extent (Figures 4C,D). These results suggested that CBM32 could increase the affinity of VxAly7B to the substrate.

We also prepared insoluble substrate alginate gel beads to investigate whether CBM32 enhances the affinity between VxAly7B and insoluble alginate gel. The recombinant proteins were incubated with alginate gel beads at 4°C for 3 h. After centrifugation, the unbound proteins in the supernatants were measured by SDS-PAGE. The results showed that recombinant VxAly7B-FL and VxAly7B-CBM could be completely combined with alginate gel beads, while VxAly7B-CM was partially combined, which proved that CBM32 could promote the combination of VxAly7B with alginate gel (Figure 4E). Simultaneously, the Langmuir-type adsorption isotherm was used to calculate the adsorption parameters to quantitatively evaluate the binding effect of VxAly7B-FL, VxAly7B-CM, and VxAly7B-CBM on the insoluble alginate gel. The maximum adsorption capacity of recombinant VxAly7B-FL on alginate gel beads (4,285 nmol/g substrate) was much higher than that of VxAly7B-CM (2,779 nmol/g substrate) but lower than that of VxAly7B-CBM (14,489 nmol/g substrate). The theoretical binding capacity of VxAly7B-CBM was the highest, which was 3.3-fold that of VxAly7B-FL and 5.2-fold that of VxAly7B-CM.

**FIGURE 5:**
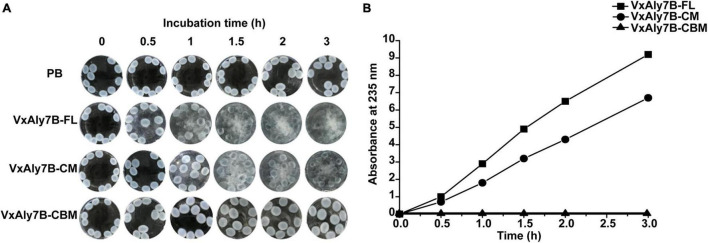
Degradation ability of CBM32 on alginate gel beads. **(A)** Catalytic effect of recombinant VxAly7B-FL, VxAly7B-CM, and VxAly7B-CBM on alginate gel beads. **(B)** Changes in the reducing sugar contents of recombinant VxAly7B- FL-, VxAly7B-CM- and VxAly7B-CBM-degraded alginate gel beads (VxAly7B-FL, square; VxAly7B-CM, circle; VxAly7B-CBM, triangle).

To prove whether CBM32 enhanced the activity of the catalytic domain of VxAly7B on insoluble substrates, the degradation process of recombinant VxAly7B-FL, VxAly7B-CM, and VxAly7B-CBM on insoluble alginate gel beads were determined. The morphological changes of the gel beads were observed and recorded every 0.5 h. The results showed that the alginate gel beads of the control group supplemented with PB did not change from 0 to 3 h (Figure 5A). The alginate gel beads for the experimental group with added recombinant VxAly7B-FL obviously swelled and broke in 1 h and were completely broken at 3 h (Figure 5A). However, the alginate gel beads with added recombinant VxAly7B-CM changed slowly and swelled and broke in 3 h (Figure 5A). Correspondingly, the alginate gel beads with added recombinant VxAly7B-CBM swelled but did not break, and their diameter swelled from 2 mm to approximately 4 mm in 3 h.

To further determine the degradation ability of the recombinant protein toward alginate gel beads, the change in reducing sugar content in the product in the reaction system was measured. With the extension of time, the reducing sugar content of the recombinant VxAly7B-FL system and the VxAly7B-CM system gradually increased, but the changes were more significant in the recombinant VxAly7B-FL system (Figure 5B). The results showed that the reducing sugar content in the recombinant VxAly7B-FL system was 1.28 times that of the VxAly7B-CM system in 3 h. Recombinant VxAly7B-CBM had no degradation ability to alginate gel beads, and the reducing sugar content was not detected (Figure 5B). The catalytic process of the recombinant enzyme on the alginate gel beads and the determination of the reducing sugar content showed that the recombinant VxAly7B-FL has a stronger ability to degrade insoluble alginate gel beads. It was found that CBM32 can promote the degradation of insoluble alginate gel. These results revealed that CBM32 might improve the degradation efficiency of VxAly7B toward alginate gel beads by swelling the alginate gel beads and concentrating the enzyme on the substrate.

## Discussion

In our study, we characterized the alginate lyase VxAly7B from *V. xiamenensis* QY104 and its truncated mutants and found that CBM32 in VxAly7B exhibits different biochemical characteristics for soluble and insoluble substrates. As an auxiliary module, the CBM domain is essential in the process of binding to insoluble substrates. The CBM54 domain from laminarinase Lic16A could bind a variety of insoluble polysaccharides but cannot bind soluble polysaccharides and oligosaccharides (Voronina and Pshennikova, 2010). CBM1 from *Th*CBM1_*Cel*7A_ and *Th*CBM1_*Cel*7B_ bound irreversibly to the insoluble substrate, thereby disrupting insoluble polysaccharide structures and leading to its amorphization (Bernardes et al., 2019). Endo-guluronate lyase from *Zobellia galactanivorans* DsiJT and Aly7D from *Saccharophagus degradans* 2–40, which contain CBM32, could effectively destroy the *Laminaria digitata* recalcitrant cell wall, while other alginate lyases without CBM32, such as PA1167 from *Pseudomonas aeruginosa* PAO1 and A1m from *Agarivorans* sp. JAM-A1m had no effect (Costa et al., 2021). Therefore, we speculate that CBM32 plays a significant role in the degradation of insoluble alginate.

**FIGURE 6:**
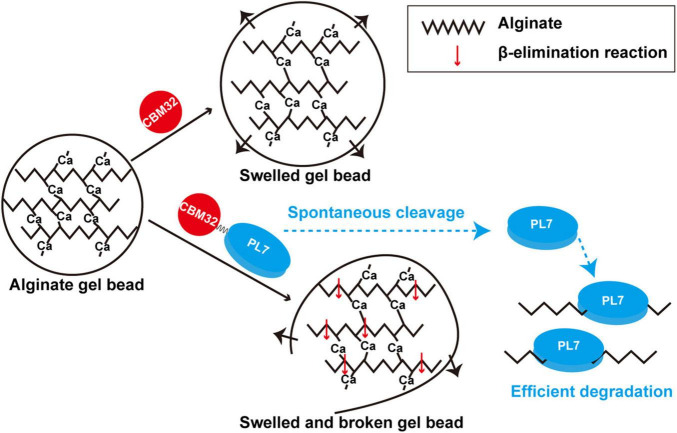
Schematic diagram of degradation modes of soluble sodium alginate and insoluble alginate gel by VxAly7B.

We used the alginate lyase VxAly7B to determine the function of CBM32 on the insoluble substrate alginate gel. High molecular weight alginate has many Ca^2+^-binding sites and can form a dense gel form with a permeable network structure of high water holding capacity, strength, and elasticity with Ca^2+^(Cao et al., 2020). The spatial structure of this Egg-box model made the alginate gel beads have no obvious morphological changes in PB (Figure 5A). By measuring the binding and degradation ability of insoluble alginate gel beads by recombinant VxAly7B-FL, VxAly7B-CM, and VxAly7B-CBM, it was found that CBM32 could efficiently bind insoluble alginate gel beads and promote the degradation of the gel beads by the PL7 catalytic domain (Figures 4E, 5, 6). This is a breakthrough in the functional identification of CBM32 in alginate lyase toward insoluble alginate gel. In addition to the interaction of Ca^2+^ with the carboxyl groups in alginate, hydrogen bonds, van der Waals forces, and/or hydrophobic interactions also contribute to the formation of gels (Kastner et al., 2012). Interference with these stable interactions might destroy the dense network structure of alginate gel. The crystal structure of CBM32 in the alginate lyase AlyQ from *Persicobacter* sp. CCB-QB2 showed that N206, D209 and T307 (corresponding to N43, D46, and T151 in CBM32 of VxAly7B) coordinate with Ca^2+^ (Sikorski et al., 2007; Sim et al., 2017; Cao et al., 2020). W129 of CBM32 in AlyB from *Vibrio spendidus* 12B01 (corresponding to W118 in CBM32 of VxAly7B) might interact with negatively charged alginate (Lyu et al., 2018). Therefore, when the recombinant VxAly7B-CBM was incubated with alginate gel beads, the potential amino acid residues of CBM32 in VxAly7B could interact with Ca^2+^ and substrate in alginate gel, which might destroy the gel structure of alginate gel beads, resulting in structural change, permeability enhancement, and morphological swell. The structure of the loose alginate gel beads might further expose the alginate group to facilitate the degradation of the substrate by the PL7 catalytic domain (Figure 6).

However, the effect of CBM32 on soluble alginate in different alginate lyases showed different characteristics, which might be due to the structural differences between different CBM32, or the complex interaction between CBM32 and the catalytic domain. CBM32 of AlyM reduced the specific activity and temperature stability of the enzyme and increased the affinity toward polyM (Yang et al., 2019), while CBM32 of Dp0100 and AlyB promoted the binding and catalysis abilities (Lyu et al., 2018; Ji et al., 2019). Compared with AlyQ*_*C*_* without CBM32, the *K*_*m*_, *k*_*cat*_ and *k*_*cat*_/*K*_*m*_ of AlyQ*_*BC*_* were increased, indicating that CBM32 in AlyQ*_*BC*_* was not conducive to the binding of the enzyme to the substrate but helped the enzyme to catalyze the substrate (Sim et al., 2017). In our study, CBM32 had no obvious effect on the optimal temperature, pH, and substrate preference of VxAly7B, but it significantly reduced the temperature stability of VxAly7B. When recombinant VxAly7B-FL and VxAly7B-CM were incubated for 1 h at 40°C, approximately 87% of the enzymatic activity of VxAly7B-CM was retained, while for VxAly7B-FL only 16% was retained (Figure 2F). Importantly, CBM32 of VxAly7B was not conducive to the binding and catalysis of soluble alginate by recombinant VxAly7B. The substrate-binding and catalytic abilities of recombinant VxAly7B-FL were both higher than those of recombinant VxAly7B-CM (Table 2). Similar results also appeared in the alginate lyase AlyH of *Marinimicrobium* sp. H1 (Yan et al., 2019). The results showed that AlyH-I without CBM32 exhibited lower *K*_*m*_ and higher *k*_*cat*_/*K*_*m*_ than AlyH-II with CBM32. Overall, CBM32 could promote the binding and catalytic abilities of VxAly7B to insoluble alginate, but it was not conducive to the degradation of soluble alginate. This feature might be closely related to the spontaneous cleavage of VxAly7B.

In addition, we purified the native alginate lyase VxAly7B from the fermentation broth of *V. xiamenensis* QY104 (Figure 1A). Spontaneous cleavage occurred during the fermentation process and was increased with time. Such cleavage of CBM32 was demonstrated in both native and recombinant VxAly7B (Figure 1D and Supplementary Figure 4). Although the mechanism of the spontaneous cleavage of VxAly7B is still unclear, we found that the reaction was slow and the increase in temperature and the extension of time will accelerate its progress (Figure 1D and Supplementary Figure 4). The internal cleavage of CBM54 of laminarinase Lic16A and chitinase ChiW is a common feature, and the spontaneous cleavage of CBM54 is essential for the combination of the catalytic module and insoluble polysaccharides (Itoh et al., 2014; Dvortsov et al., 2018). The N-terminal extension (CBM_4_9 module) of AlyA and aly-SJ02 helps the protein to be folded correctly and then breaks, which is a common phenomenon of PL18 family alginate lyases (Matsushima et al., 2010; Dong et al., 2014). Unlike the internal cleavage of some other CBMs, the spontaneous cleavage of CBM32 is not a uniform feature in the family. Most of CBM32 exists stably in alginate lyase (Lyu et al., 2018; Yan et al., 2019), only a few of CBM32 spontaneously cleaved from the alginate lyase, such as alginate lyase in *Vibrio* sp. QY102 (Sun et al., 2019) and VxAly7B in *V. xiamenensis* QY104. After the disordered region between the two modules was broken, the activity of the enzyme only containing the catalytic domain increased (Figure 6). The characteristics of CBM32 in different alginate lyases might be closely related to the growth environment of the strain or enzyme and its biological function. We speculated that in the early growth of *V. xiamenensis* QY104, to accelerate the expansion and degradation of the substrate, full-length VxAly7B utilizes CBM32 to efficiently bind insoluble alginate substrates such as laminarin. With the loosening of the substrate structure, CBM32 spontaneously cleaved, and a catalytic domain lacking CBM32 was obtained. The catalytic domain has higher activity and stability for soluble substrates and deeply degrades alginate to effectively produce a carbon source for bacterial growth. As an efficient catalytic tool, VxAly7B can not only degrade insoluble alginate but also quickly utilize soluble alginate, thus may assist the rapid degradation of brown algae.

## Conclusion

In this study, we used the alginate lyase VxAly7B to determine the function of CBM32 on the soluble sodium alginate and insoluble alginate gel. The results of the activity analysis showed that CBM32 was not conducive to the temperature stability of VxAly7B and its binding and catalytic ability to soluble sodium alginate. By measuring the binding and degradation ability of insoluble alginate gel beads, we found that CBM32 could efficiently bind insoluble alginate gel beads and promote the degradation of the gel beads by the PL7 catalytic domain. As an efficient catalytic tool, VxAly7B can not only degrade insoluble alginate but also quickly utilize soluble alginate, which could promote our understanding of the mechanism of alginate polysaccharide metabolism.

## Data Availability Statement

The datasets presented in this study can be found in online repositories. The names of the repository/repositories and accession number(s) can be found below: https://www.ncbi.nlm.nih.gov/genbank/, AGL78596.1.

## Author Contributions

LT: conceptualization, data curation, validation, methodology, visualization, roles and writing—original draft, and writing—review and editing. EG and LZ: conceptualization, data curation, and methodology. YW, SG, and MB: data curation and validation. FH: conceptualization, funding acquisition, methodology, supervision, and writing—review and editing. WY: supervision and writing—review and editing. All authors contributed to the article and approved the submitted version.

## Conflict of Interest

The authors declare that the research was conducted in the absence of any commercial or financial relationships that could be construed as a potential conflict of interest.

## Publisher’s Note

All claims expressed in this article are solely those of the authors and do not necessarily represent those of their affiliated organizations, or those of the publisher, the editors and the reviewers. Any product that may be evaluated in this article, or claim that may be made by its manufacturer, is not guaranteed or endorsed by the publisher.

## Footnotes

1
http://www.cazy.org/

2
http://espript.ibcp.fr/ESPript/cgi-bin/ESPript.cgi
