# Supplementary material for: The Function of CBM32 in Alginate Lyase VxAly7B on the Activity on Both Soluble Sodium Alginate and Alginate Gel
Source: Front Microbiol. 2022 Jan 7;12:798819. doi: 10.3389/fmicb.2021.798819 (PMC8776709; doi:10.3389/fmicb.2021.798819)
Supplement: Supplementary file 1 [file Data_Sheet_1.docx]

Supplementary material

# The function of CBM32 domain in alginate lyase VxAly7B on the activity on both soluble sodium alginate and alginate gel

Luyao Tang^1,2,3,4†^, Enwen Guo^1,2,3,4†^, Lan Zhang^1,2,3,4†^, Ying Wang^1,2,3,4^, Shan Gao^1,2,3,4^, Mengmeng Bao^1,2,3,4^, Feng Han^1,2,3,4^*, Wengong Yu^1,2,3,4^*

**Table S1 PCR primers for the recombinant VxAly7B and its** **truncated mutants**

| **Primer name** | **Primer sequences (5’-3')** |
| --- | --- |
| VxAly7B-FL-F | CATATGGATACACTGCCGATCCT |
| VxAly7B-FL-R | CTCGAGGTAAGAATAATTTGAATGTTCG |
| VxAly7B-CM-F | CATATGATTCTTGGTAGTAACAAGAAC |
| VxAly7B-CM-R | CTCGAGGTAAGAATAATTTGAATGTTCG |
| VxAly7B-CBM-F | CATATGGATACACTGCCGATCCT |
| VxAly7B-CBM-R | CTCGAGATTGACTTCGGTAATATTGGTC |

**Table S2 Protein sequencing of native alginate lyase VxAly7B**

| **Sequencing method** | **Amino acid sequences** |
| --- | --- |
| N-terminal sequencing | ILGSNYKNYGLLDAKKPP |
| Mass spectrometry-based  de novo sequencing of intermediate peptide | ALYFAHESSK  VLGQLHAK  TLAVNHVTTTTSDSK  IYYHKLPENEK  EAADSWPVYELDVVADSLTVTLR |

**
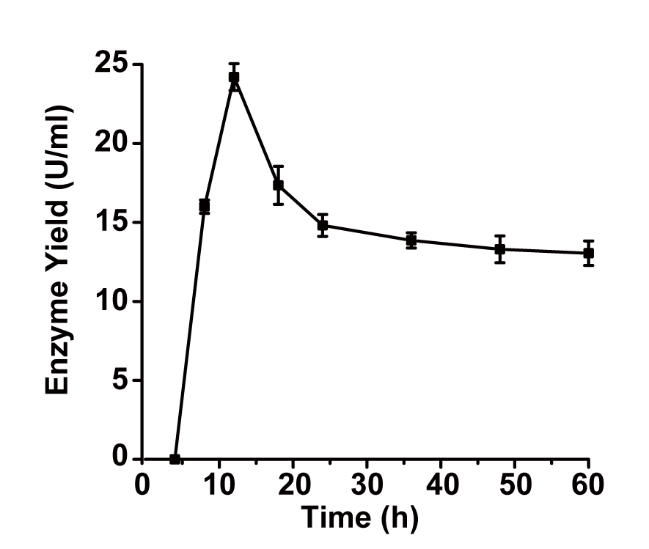
**

**Figure S1 Enzyme yield of *Vibrio xiamenensis* QY104.**


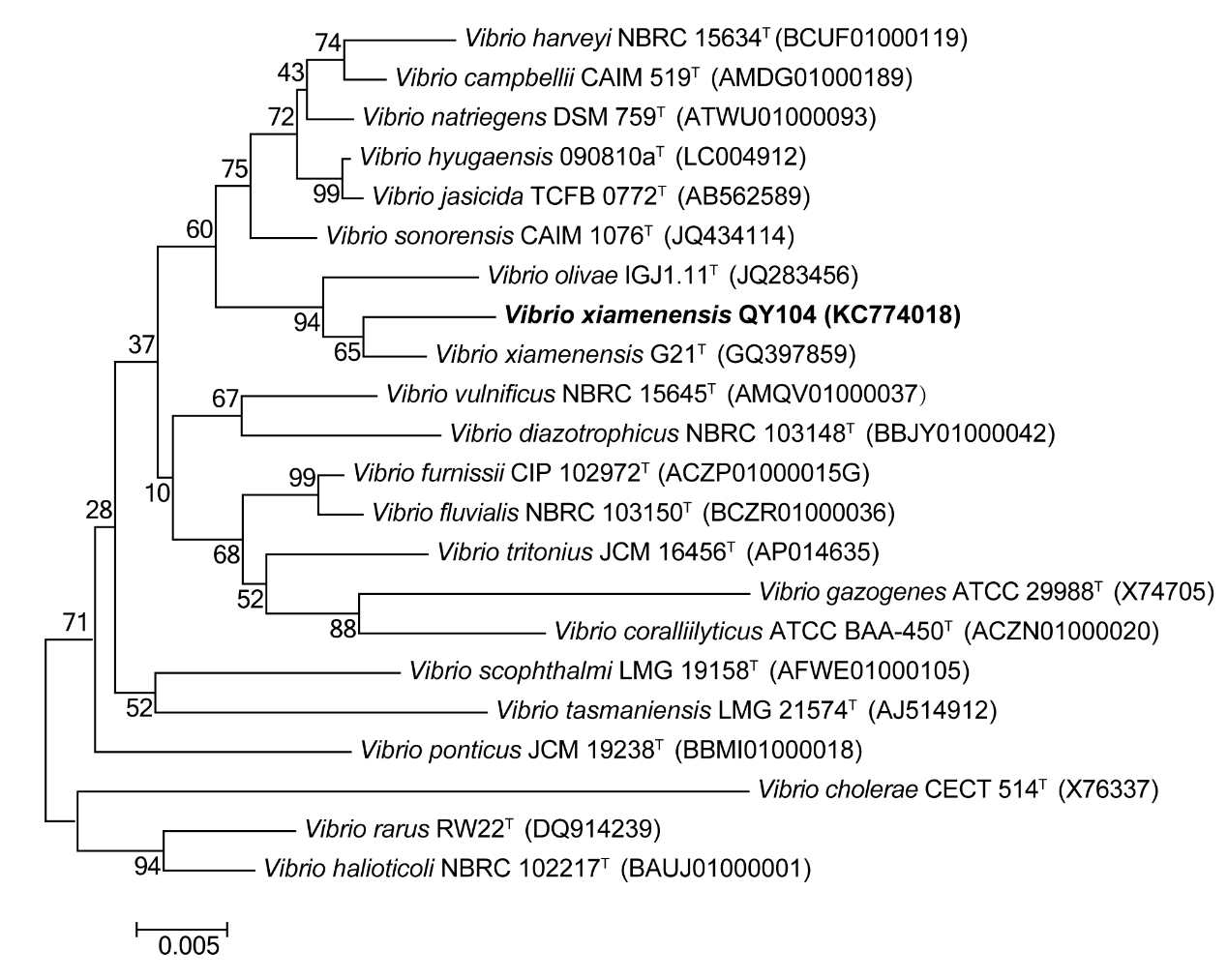


**Figure S2 Phylogenetic analysis of *V. xiamenensis* QY104.** The phylogenetic tree was constructed with MEGA 7.0 using the neighbour-joining method. Bootstrap values were expressed based on 1000 replications, the numbers represent bootstrap values (confidence limits) representing the substitution frequencies per amino acid residue. The sequences from this study are shown in bold.


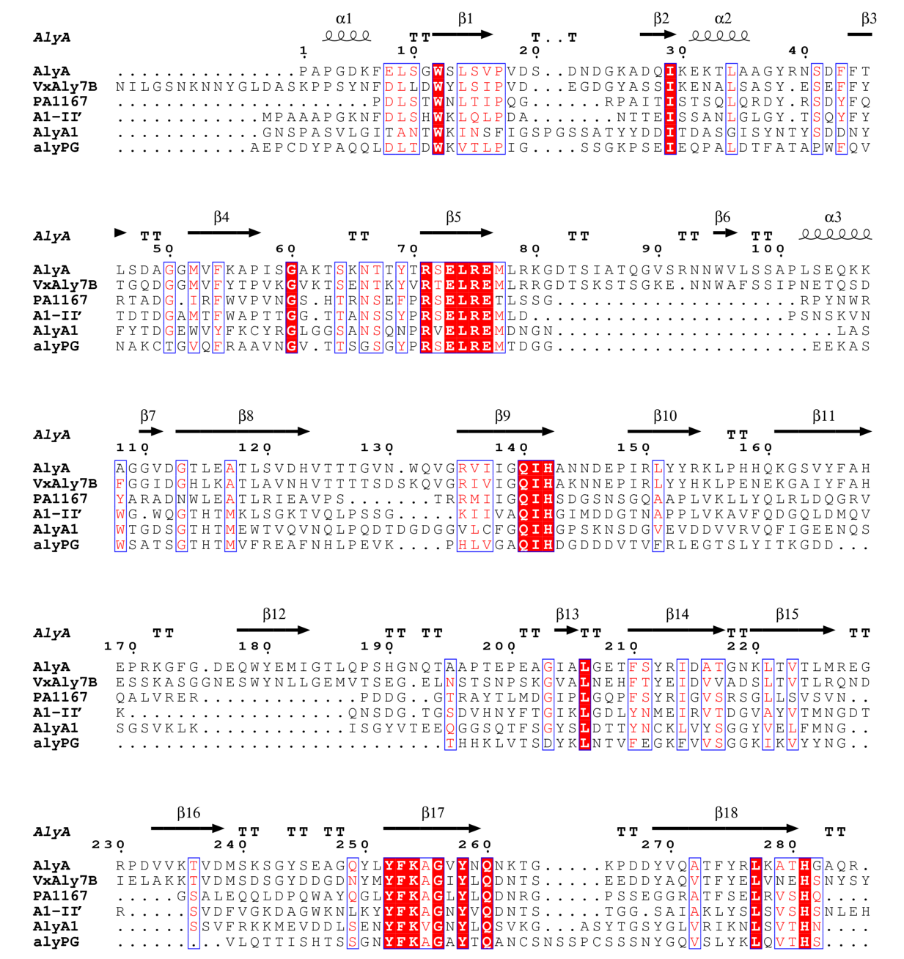


**Figure S3 Multiple amino acid sequence alignment of VxAly7B**. Sequence alignment of VxAly7B with some crystallized PL7 enzymes. The secondary structure elements shown above are referenced according to AlyA. AlyA, from *Klebsiella pneumoniae subsp. aerogenes* (AAA25049); PA1167, from *Pseudomonas aeruginosa* PAO1 (AAG04556); A1-II’, from *Sphingomonas* sp. A1 (BAD16656); AlyA1, from *Zobellia galactanivorans* DsiJT (CAZ95239); alyPG, from *Corynebacterium* sp. ALY-1 (BAA83339).


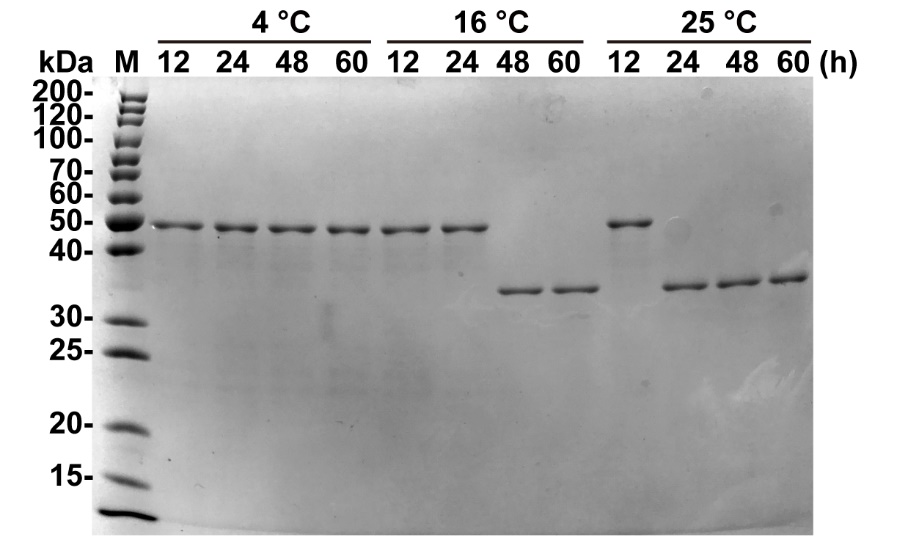


**Figure S4 Spontaneous cleavage of recombinant VxAly7B-FL.** Lane M, molecular weight markers.

**
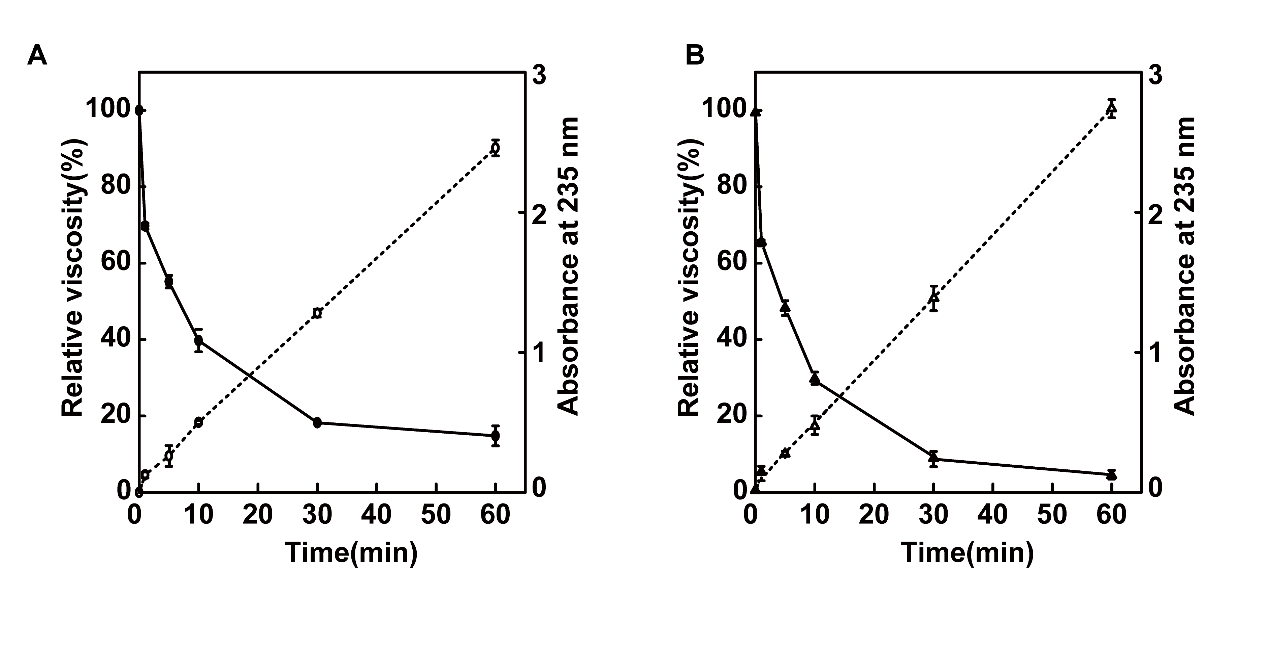
**

**Figure S5 Modes of action of recombinant VxAly7B-FL (A) and VxAly7B-CM (B).** The reaction was conducted at 30 °C using 3 mg/mL sodium alginate and 3 U/mL recombinant VxAly7B-FL or VxAly7B-CM. The changes in absorbance at 235 nm (dotted line) and reduction in viscosity (solid line) were measured.
